# Vertebral Cervical Fusion in Individuals with and without Open Bite: A Comparative Matched Study

**DOI:** 10.4317/jced.63888

**Published:** 2026-03-30

**Authors:** Lucecita Angeles-Estrella, Jhoana Mercedes Llaguno-Rubio, Gustavo Adolf Fiori-Chincaro, Luis Ernesto Arriola-Guillén

**Affiliations:** 1 Division of Oral and Maxillofacial Radiology, Faculty of Dentistry, Centro Universitário do Norte de São Paulo (UNORTE), São Paulo, Brazil; 2 Associate Professor, Division of Oral Radiology, Instituto Latinoamericano de Altos Estudios en Estomatología (ILAE), Lima, Peru; 3 Associate Professor, Division of Oral Radiology, Instituto Latinoamericano de Altos Estudios en Estomatología (ILAE), Lima, Peru; 4 Ph.D. and Associate Professor, Division of Orthodontics, Faculty of Dentistry, Universidad Científica del Sur, Lima, Peru

## Abstract

**Background:**

This study aims to evaluate the presence of cervical vertebral fusion in individuals with an open bite compared to those without, matched by skeletal relationship, age, and sex.

**Material and Methods:**

This matched-comparative study analyzed 234 lateral head radiographs, dividing the subjects into two groups. The open-bite group consisted of 117 patients (69 women and 48 men; average age 24.05 ± 12.5 years), while the control group included an equal number of individuals with a similar gender ratio (average age 23.79 ± 11.95 years). Both groups were matched based on their skeletal relationship, specifically the ANB angle. A trained and calibrated radiologist conducted a visual assessment of the cervical spine using lateral cephalometric radiographs for each subject. The study evaluated the presence of simple fusion and block fusion. Statistical analyses were performed using the Chi-square test and binary logistic regression (p &lt; 0.05).

**Results:**

The incidence of simple fusion was higher in the control group (35%) compared to the open-bite group (26.5%). A small number of cases (2.6%) of block fusion were found only in the anterior open-bite group. However, these differences did not reach statistical significance (p = 0.096). Additionally, no significant influences were identified regarding sex, age, group, or ANB angle (p &gt; 0.05).

**Conclusions:**

There were no differences in the overall prevalence of cervical fusions between individuals with anterior open bite and those without. However, the finding that block fusions occurred exclusively in the open-bite group underscores the importance of evaluating the cervical spine, as it may affect the individual's head position.

## Introduction

The relationship between cervical vertebral structure and facial skeletal changes has attracted increasing attention in the dental and orthodontic literature. Several studies suggest that cervical vertebral anomalies, such as fusions and other morphological changes, may be associated with various types of skeletal malocclusions. The influence may explain this association between these anomalies and craniocervical posture, muscular balance, and craniofacial development (1 - 5). Anterior open bite is a complex malocclusion characterized by the lack of vertical contact between the maxillary and mandibular anterior teeth, which compromises both masticatory function and facial aesthetics (6). Its etiology is multifactorial, involving persistent oral habits, genetic factors, respiratory issues, and changes in skeletal growth patterns (7 , 8). Recent studies report a prevalence of anterior open bite ranging from 15% to 20% in pediatric and adolescent populations, emphasizing its clinical significance and the need for further investigation into associated factors (9). Cervical fusions, as extensively described by Sonnesen and Kjaer (1 , 4 , 5), are developmental anomalies that can affect the craniocervical relationship and mandibular position. It has been suggested that abnormal cervical morphology may affect head posture and the direction of facial growth, potentially resulting in skeletal patterns associated with malocclusions, such as anterior open bite (10 - 12). Recent research supports this hypothesis, indicating that structural variations in the cervical vertebrae may influence the orientation of the mandibular plane and the patterns of the facial skeleton (13 , 14). However, the available evidence remains inconclusive. For instance, Kim et al. (15) found associations between cervical morphology and head posture but did not identify cervical fusion as a distinguishing factor among different subtypes, such as open bite. Similarly, other studies have reported no statistically significant distribution of cervical anomalies across various types of malocclusions, nor consistent associations with variables such as age, sex, skeletal classes, or facial types (16 , 17). Likewise, Bebnowski et al. (18) questioned the diagnostic reliability of lateral cephalometric radiographs, demonstrating that some fusions identified on these images could not be confirmed by computed tomography. Historical literature indicates a strong association between vertebral anomalies and malocclusions; however, current evidence is mixed and limited, warranting clinical caution. This lack of a direct association suggests that, in populations without related syndromes, variations in cervical morphology may be incidental findings rather than contributing factors in the development of open bite cases. In this context, the present study aims to compare the prevalence of cervical vertebral fusion in individuals with and without anterior open bite, matched by skeletal relationship, age, and sex. This approach seeks to enhance our understanding of the relationship between cervical structures and dentoskeletal alterations, providing evidence to support orthodontic diagnosis and promote a more precise, interdisciplinary therapeutic approach.

## Materials and Methods

- Study design and Ethical Approval This was a matched-comparative study approved by the Centro Universitário do Norte de São Paulo (UNORTE), registration number: 002/2026. This study complied with the Declaration of Helsinki and the institution's relevant ethical regulations. Permission to access and use lateral skull radiographs was obtained via a formal letter addressed to the directors of the two private dental imaging diagnostic centers involved in the research. - Sample size calculation The study population consisted of digital lateral cephalometric radiographs of patients aged 6 years or older who were treated at two dental imaging diagnostic centers in Santiago de los Caballeros, Dominican Republic. The radiographs were collected between August 2009 and August 2025 and sourced from the institutional digital archives of both centers. A total of 234 lateral head radiographs were analyzed, with subjects divided into two groups. The open-bite group comprised 117 patients (69 women and 48 men), with an average age of 24.05 ± 12.5 years. The control group consisted of an equal number of individuals with a similar gender ratio, with an average age of 23.79 ± 11.95 years. Both groups were matched based on their skeletal relationship, specifically the ANB angle. Sample size was calculated using a comparison of two prevalence proportions for two study groups: Individuals with anterior open bite and patients without anterior open bite. This calculation was performed using the Fisterra software (www.fisterra.com), with a confidence level of 95% and a statistical power of 80% to detect statistically significant differences between the two groups. A fusion proportion of 40.5% was expected in the control group, and 25% in the open-bite group, based on data from a pilot study. A minimum of 117 radiographs by group were required. The selection of radiographs was conducted after verifying compliance with the study's inclusion and exclusion criteria and ensuring the diagnostic quality of the images. This process was crucial for the validity and reliability of the analyzed information. - Selection Criteria For the open bite group, digital lateral cephalometric radiographs of individuals of both sexes exhibiting an anterior open bite greater than 0 mm were included. Only images of adequate diagnostic quality, in terms of sharpness and contrast, were considered suitable for proper visualization and evaluation of the first five cervical vertebrae (C1-C5). Radiographs of individuals with a history of cervical trauma, the presence of orthodontic appliances at the time of acquisition, or any bone pathologies that could alter cervical vertebral morphology were excluded. For the control group, digital lateral cephalometric radiographs of patients of both sexes who did not present an anterior open bite were included. As in the study group, only images of adequate diagnostic quality that allowed evaluation of the first five cervical vertebrae (C1-C5) were selected. Exclusion criteria were the same as those for the study group. - Image Acquisition The images were obtained using a Planmeca ProMax 2D unit with a frequency range of 80-150 kHz, selectable pixel sizes of 48/96/144 m, and exposure times of 2.7-16 seconds. - Image Evaluation The collected images were evaluated using the Windows Image Viewer on a computer equipped with an Apple Studio Display, with a screen resolution of 5120 × 2880 pixels and an approximate visible surface area of 19.9 dm². The assessment was conducted in a controlled environment measuring 4 × 3 meters, with dim natural light estimated at 25 lux, during morning hours between 9:00 and 11:00 a.m. The evaluator was seated approximately 40 cm from the monitor in an ergonomic chair, maintaining a spinal axis angle of 60°-90° to standardize observation conditions. A pilot test was conducted to train and calibrate the evaluator. The findings were reviewed and confirmed by two radiology specialists. The morphological characteristics of the cervical spine were assessed according to the criteria proposed by Sonnesen and Sandham, which classify cervical vertebral anomalies as either simple fusion (union of two adjacent vertebrae) or block fusion (union of three or more cervical vertebrae). The independent variable was the presence and type of cervical fusion, while the dependent variables included sex, age, and the patients' skeletal pattern. The skeletal pattern was determined using the ANB angle, obtained via the WebCeph digital platform (webceph.com) from the digitized lateral cephalometric radiographs. Collected data were recorded in a Microsoft Excel spreadsheet, ensuring participant anonymity through image coding. - Statistical analysis Statistical analysis was conducted using IBM SPSS Statistics version 19 (IBM Corp., NY, USA). First, a descriptive analysis of the sociodemographic and cephalometric variables in the sample was performed. To compare the anterior open-bite group with the control group, we assessed age, sex, and skeletal relationship using inferential statistical tests. This analysis confirmed that there were no statistically significant differences between the groups, assessed through t-tests, exact Fisher tests, and chi-square tests. Next, we compared the prevalence of cervical vertebral fusion and its subtypes (absence of fusion, simple fusion, and block fusion) between the two groups using the chi-square test. Finally, we conducted a binary logistic regression analysis to evaluate the influence of independent variables-sex, age, study group (anterior open bite vs. control), and ANB angle-on the presence of cervical vertebral fusion, with p &lt; 0.05 set as the threshold for statistical significance.

## Results

Table 1 presents the initial characteristics of the sample, showing agreement between the two groups regarding age (p=0.783), sex (p=1.000), and skeletal relationship (p=0.990).

Table 1

Table 2 outlines the relationship between the presence of an open bite and the occurrence of vertebral fusion.

Table 2

Both groups showed incomplete vertebral fusion, with 70.9% in the open-bite group and 65% in the control group. The incidence of simple fusion was higher in the control group, at 35%, compared to 26.5% in the open-bite group. Block fusion was observed in only a few cases (2.6%) within the open-bite group (p=0.096). Finally, Table 3 presents a binary logistic regression analysis to assess whether predictor variables influence the occurrence of vertebral fusion. Our analysis revealed no significant impact of sex, age, group, or ANB angle.

Table 3

1

**Figure 1:**
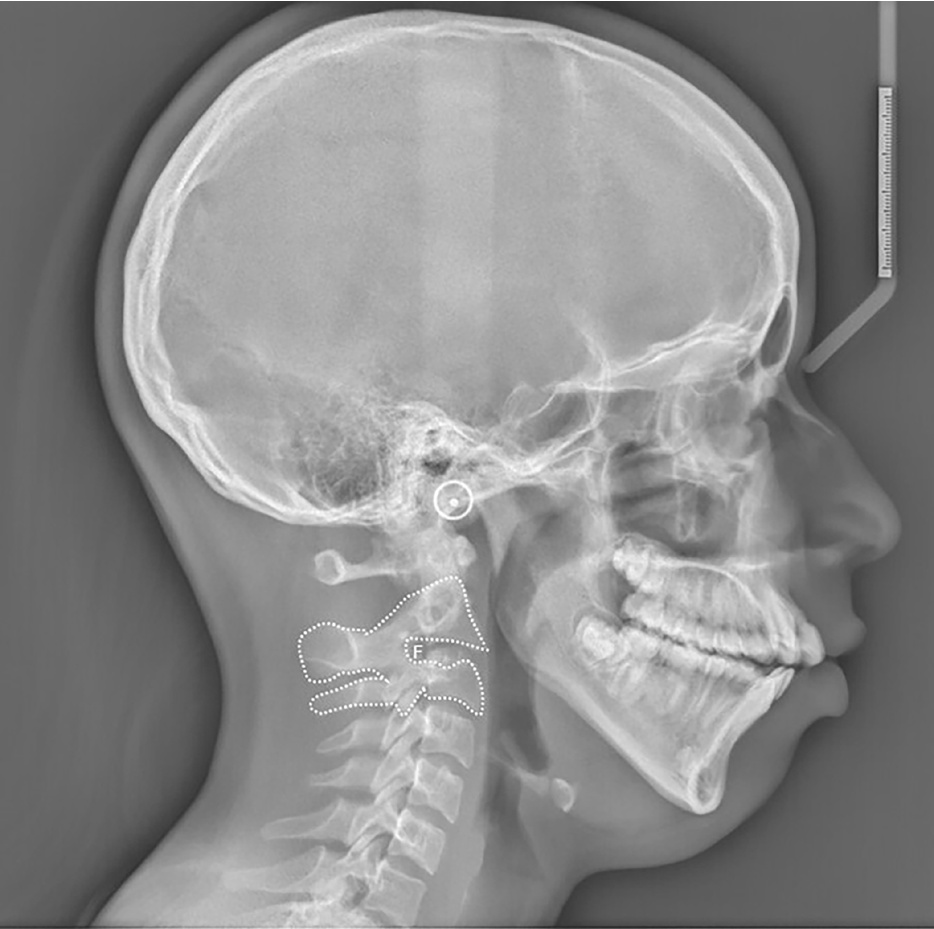
Lateral cephalometric skull radiograph demonstrating the fusion (F) between the C2 and C3 vertebrae.

## Discussion

This study aimed to evaluate the presence of cervical fusion in individuals matched for skeletal relationship, age, and sex, comparing those with an anterior open bite to those without one. We used lateral radiographs from a population in Santiago de los Caballeros, Dominican Republic. Although previous literature has suggested a potential relationship between cervical spine morphology and dentoskeletal alterations, our findings did not show a statistically significant association between anterior open bite and cervical vertebral fusions (p = 0.096). This indicates that these conditions do not necessarily occur together in the population we studied (1 - 5). When analyzing the overall prevalence of cervical fusion among patients, it was observed that most individuals in both groups did not exhibit any signs of cervical fusion. Specifically, 70.9% of patients in the open-bite group and 65.0% in the control group had no cervical fusion. These findings slightly contrast with those reported by Sonnesen and Kjaer (1 , 5 , 10), who identified significant associations between cervical anomalies and severe skeletal malocclusions, such as anterior open bite. However, their research primarily focused on Scandinavian populations (1 , 5 , 10), indicating that ethnic and demographic factors may influence the occurrence of these anomalies. A notable finding in this study was that block fusions, although rare, were exclusively observed in the anterior open-bite group (2.6%); none were found in the control group, but this was not statistically significant. Despite several authors hypothesizing that complex cervical anomalies may be linked to altered vertical facial growth patterns and increased instability of craniocervical posture (1 , 4 , 5 , 10), our results do not provide evidence for a universal association. In contrast, simple fusion was observed more frequently in the control group (35.0%) than in the open-bite group (26.5%). This finding diverges from earlier studies that reported a higher prevalence of vertebral anomalies in individuals with specific skeletal malocclusions (10 - 13 , 15). This discrepancy may be attributed to the multifactorial nature of anterior open bite, which involves not only skeletal elements but also persistent oral habits, respiratory issues, and functional components that may not directly correlate with cervical vertebral morphology (6 - 9). Among the predictor variables analyzed, the binary logistic regression model did not identify sex, age, study group, or ANB angle as significantly associated with cervical vertebral fusion. These findings align with recent research that also failed to establish consistent associations between cervical anomalies and skeletal classes, sex, or patient age (16 - 18 , 20). Specifically, studies by Ostovarrad et al. (20) and Kamak and Yildirim (21) reported a similar distribution of cervical anomalies across different facial and skeletal patterns, suggesting that these variations may function as anatomical differences independent of the maxillomandibular sagittal pattern. Additionally, Bebnowski et al. (22) noted that assessing cervical fusions using lateral cephalometric radiographs may be constrained by the overlapping of anatomical structures, making it challenging to differentiate true from apparent fusions. This methodological limitation is particularly relevant when interpreting the results of this study, as reliance on two-dimensional images may lead to underestimation or overestimation of certain vertebral anomalies. Although classical literature has suggested a strong correlation between vertebral anomalies and malocclusions, contemporary evidence yields heterogeneous results, warranting clinical caution. Our findings, which showed no statistically significant differences in the prevalence of cervical fusion between the open-bite and control groups, are consistent with reports from researchers who, after evaluating large samples, concluded that there is no direct effect of skeletal malocclusion type on the etiology of cervical vertebral anomalies (23). This lack of a direct association suggests that, in populations without associated syndromes, morphological variations may represent incidental findings rather than determining etiological factors. Nevertheless, these findings should be contrasted with studies using three-dimensional diagnostic methods. Recent investigations using cone-beam computed tomography have reported significant differences, with a higher frequency of dehiscences and fusions in skeletal Class II and III patients than in Class I patients (24). This discrepancy between two-dimensional and three-dimensional studies may explain the variability in prevalence reports, highlighting the limitations of conventional lateral radiography in detecting subtle anomalies. Additionally, the genetic and embryological components appear to play a more predominant role in conditions of greater severity. For example, a prevalence of cervical anomalies of up to 62% has been documented in patients with cleft lip and palate, a figure significantly higher than that observed in control groups (25). This finding indicates that the association is more robust in the presence of complex congenital disabilities than in isolated malocclusions. Finally, the discussion should extend beyond static morphology to also incorporate functional and demographic aspects. Recent reviews highlight the importance of considering craniocervical posture and its reciprocal influence on occlusion (26). Furthermore, there may be a connection between alterations in the craniocervical angle and temporomandibular disorders (27). It is also crucial to recognize ethnic differences in craniofacial and vertebral morphology when applying research findings from European or Asian populations to groups such as those studied in the Dominican Republic (28). Ongoing research continues to investigate these multifactorial correlations, including the relationships between pain and altered posture (29 - 31). Future studies should adopt three-dimensional approaches and be stratified by ethnic origin to improve our understanding of these associations. Limitations Among the limitations of this study, its retrospective design and the exclusive use of lateral skull radiographs should be considered, as these factors preclude a detailed three-dimensional assessment of the cervical vertebrae. However, adequate sample size, standardized image evaluation, and homogeneity of groups in terms of age, sex, and skeletal relationships strengthen the validity of the findings and allow for a cautious interpretation of the absence of statistically significant associations in this specific population.

## Conclusions

The overall prevalence of cervical fusions was similar between individuals with anterior open bite and those without it. However, the observation that block fusions were found only in the open-bite group highlights the need to evaluate the cervical spine, as this may influence the individual's head position.

## Figures and Tables

**Table 1:** Initial sample characteristics by group.

Group	n	(Age)Mean	SD	p*		
Open bite	117	24.05	12.50	0.873		
Control	117	23.79	11.95			
Group		Female	Male	Total	p**	
Open bite	n	69	48	117	1.000	
	%	59.0	41.0	100		
Control	n	69	48	117		
	%	59.0	41.0	100		
Total	n	138	96	234		
	%	59.0	41.0	100		
Group		Class I	Class II	Class III	Total	p***
Open bite	n	38	73	6	117	0.990
	%	32.5	62.4	5.1	100	
Control	n	37	74	6	117	
	%	31.6	63.2	5.1	100	
Total	n	75	147	12	234	
	%	32.1	5.1	5.1	100	

*T test, ** Exact Fisher test, *** Chi square test

**Table 2:** Association Between Open Bite and the Occurrence of Cervical Vertebrae Fusion.

Group		Absent	Simple Fusion	Blok Fusion	Total	p
Open bite	n	83	31	3	117	0.096
	%	70.9	26.5	2.6	100.0	
Control	n	76	41	0	117	
	%	65.0	35.0	0.0	100.0	
Total	n	159	72	3	234	
	%	67.9	30.8	1.3	100.0	

Chi-square test

**Table 3:** Analysis using binary logistic regression to determine whether predictor variables affect the occurrence of vertebral fusion.

Predictor variables	p	Exp(B)	C.I. To 95% for B	
			Lower	Upper
Age	0.258	1.013	0.990	1.036
Female	---	---	---	---
Male	0.564	0.844	0.474	1.502
Control group	---	---		---
Open bite group	0.320	0.756	0.435	1.313
ANB	0.610	1.030	0.921	1.151

3

## Data Availability

The data supporting the findings of this study are available from the corresponding author upon reasonable request.

## References

[1] Sonnesen L, Kjaer I (2008). Cervical column morphology in patients with skeletal open bite. Orthod Craniofac Res.

[2] Sandham A (1986). Cervical vertebral anomalies in cleft lip and palate. Cleft Palate J.

[3] Kim JR, Jo JH, Chung JW, Park JW (2020). Upper cervical spine abnormalities as a radiographic index in the diagnosis and treatment of temporomandibular disorders. Oral Surg Oral Med Oral Pathol Oral Radiol.

[4] Sonnesen L, Kjaer I (2007). Cervical vertebral body fusions in patients with skeletal deep bite. Eur J Orthod.

[5] Sonnesen L (2010). Associations between the cervical vertebral column and craniofacial morphology. Int J Dent.

[6] Galletto L, Urbaniak J, Subtelny JD (1990). Adult anterior open bite. Am J Orthod Dentofacial Orthop.

[7] Avrella MT, Zimmermann DR, Andriani JSP, Santos PS, Barasuol JC (2022). Prevalence of anterior open bite in children and adolescents: a systematic review and meta-analysis. Eur Arch Paediatr Dent.

[8] Akshaya K, Jain RK, Prasad AS (2022). Assessment of anterior open bite prevalence in children visiting a dental hospital: a retrospective evaluation. J Adv Pharm Technol Res.

[9] Zhou Z, Liu F, Shen S, Shang L, Wang X (2016). Prevalence of and factors affecting malocclusion in primary dentition among children in Xi’an, China. BMC Oral Health.

[10] Sonnesen L, Pedersen CE, Kjaer I (2007). Cervical column morphology related to head posture, cranial base angle, and condylar malformation. Eur J Orthod.

[11] Sonnesen L, Kjaer I (2008). Anomalies of the cervical vertebrae in patients with skeletal Class II malocclusion and horizontal maxillary overjet. Am J Orthod Dentofacial Orthop.

[12] Sonnesen L, Kjaer I (2007). Cervical column morphology in patients with skeletal Class III malocclusion and mandibular overjet. Am J Orthod Dentofacial Orthop.

[13] Arntsen T, Sonnesen L (2011). Cervical vertebral column morphology related to craniofacial morphology and head posture in preorthodontic children with Class II malocclusion and horizontal maxillary overjet. Am J Orthod Dentofacial Orthop.

[14] Sonnesen L, Petri N, Kjaer I, Svanholt P (2008). Cervical column morphology in adult patients with obstructive sleep apnoea. Eur J Orthod.

[15] Kim P, Sarauw MT, Sonnesen L (2014). Cervical vertebral column morphology and head posture in preorthodontic patients with anterior open bite. Am J Orthod Dentofacial Orthop.

[16] Nogueira Fialho MP, Pinzan-Vercelino CR, Nogueira RP, Gurgel JA (2014). Relationship between facial morphology, anterior open bite and non-nutritive sucking habits during the primary dentition stage. Dental Press J Orthod.

[17] Watanabe M, Yamaguchi T, Maki K (2010). Cervical vertebra morphology in different skeletal classes: A Three-Dimensional Computed Tomography Evaluation. The Angle Orthodontist.

[18] Santos PL, Castro MF, Castro RG, Ferreira RI (2020). Relationship between cervical vertebral anomalies and craniofacial patterns in orthodontic patients. Angle Orthod.

[19] Faruqui S, Fida M, Shaikh A (2014). Cervical vertebral anomalies in skeletal malocclusions: a cross-sectional study on orthodontic patients at the Aga Khan University Hospital, Pakistan. Indian J Dent Res.

[20] Kamak H, Yildirim E (2015). The distribution of cervical vertebrae anomalies among dental malocclusions. J Craniovertebr Junction Spine.

[21] Ostovarrad F, Faghani M, Yousefi Z, Tadayoni Z, Tofangchiha M, Caputo I (2024). Cephalometric evaluation of the relationship between cervical vertebral morphology and anomalies and the cranial base angle in different facial types and skeletal classes. Odovtos Int J Dent Sci.

[22] Bebnowski D, Hanggi MP, Markic G, Roos M, Peltomaki T (2012). Cervical vertebrae anomalies in subjects with Class II malocclusion assessed by lateral cephalogram and cone beam computed tomography. Eur J Orthod.

[23] Adisen SR, Adisen MZ, Ozdiler FE (2020). The evaluation of the relationship between cervical vertebral anomalies with skeletal malocclusion types and upper airway dimensions. Cranio.

[24] Aranitasi L, Tarazona B, Zamora N, Gandía JL, Paredes V (2017). Influence of skeletal class in the morphology of cervical vertebrae: A study using cone beam computed tomography. Angle Orthod.

[25] Ajami S, Dehghanpoor S, Tabibi SS, Movahhedian N (2025). Prevalence of upper cervical vertebral anomalies in children with non-syndromic cleft lip and/or palate in comparison with children without cleft in Iranian population. BMC Oral Health.

[26] Kui A, Bereanu A, Condor AM, Pop D, Buduru S, Labunet A (2024). Craniocervical Posture and Malocclusion: A Comprehensive Literature Review of Interdisciplinary Insights and Implications. Medicina (Kaunas).

[27] Di Giacomo P, Ferrara V, Accivile E, Ferrato G, Polimeni A, Di Paolo C (2018). Relationship between Cervical Spine and Skeletal Class II in Subjects with and without Temporomandibular Disorders. Pain Res Manag.

[28] Oh E, Ahn SJ, Sonnesen L (2018). Ethnic differences in craniofacial and upper spine morphology in children with skeletal Class II malocclusion. Angle Orthod.

[29] Anusuya V, Sharan J, Jena AK (2020). A study of cervical vertebra anomalies among individuals with different sagittal and vertical facial growth patterns. J Craniovertebr Junction Spine.

[30] Lekaviciute R, Sopagiene D, Trakiniene G, Lopatiene K (2025). The relationship between vertical malocclusions and ossification changes in the cranial base and upper cervical spine. Sci Rep.

[31] Peng H, Liu W, Yang L, Yan P, Zhong W, Gao X (2024). Craniocervical posture in patients with skeletal malocclusion and its correlation with craniofacial morphology during different growth periods. Sci Rep.

